# Slow Releasing CO Donor Modulates Susceptibility to Seizures in Rats with Chronic Prostatitis/Chronic Pelvic Pain Syndrome: Behavioral and EEG Study

**DOI:** 10.3390/medicina62010015

**Published:** 2025-12-21

**Authors:** Nikola Šutulović, Neriman Ezgin, Emilija Djuric, Milena Vesković, Dušan Mladenović, Zorica Nestorović, Aleksandra Rašić-Marković, Olivera Stanojlović, Dragan Hrnčić

**Affiliations:** 1 Institute of Medical Physiology “Richard Burian”, Faculty of Medicine, University of Belgrade, 11000 Belgrade, Serbia; 2 Department of Biotechnology, Institute of Natural and Applied Sciences, Cukurova University, Adana 01330, Turkey; 3 Institute of Pathophysiology “Ljubodrag Buba Mihailovic”, Faculty of Medicine, University of Belgrade, 11000 Belgrade, Serbia; 4 Institute of Biophysics, Faculty of Medicine, University of Belgrade, 11000 Belgrade, Serbia

**Keywords:** seizures, prostatitis, carbon monoxide, lindane, EEG

## Abstract

*Background and Objectives*: Chronic prostatitis/chronic pelvic pain syndrome (CP/CPPS) is a complex disease that involves changes in multiple organs and even the central nervous system (CNS). CP/CPPS may elevate seizure risk via neuroinflammatory mechanisms within the CNS. Neuroprotective effects of CO-releasing molecules (CORMs) were demonstrated in inflammation-driven conditions, while CORMs potential to ameliorate seizure susceptibility in inflammatory states, like CP/CPPS, remains unclear. Therefore, we investigated effects of CORM-A1 on susceptibility to lindane–induced seizures in rats with CP/CPPS through behavioral and electroencephalographic (EEG) study. *Materials and Methods*: Wistar rats were divided into four groups (n = 8/group): Sham-PBS, Sham-CORM, CP/CPPS-PBS and CP/CPPS-CORM. The CP/CPPS model was created by injection of 3% λ-carrageenan and its development assessed by mechanical pain threshold. CORM-A1 (2 mg/kg/day, i.p.) or vehicle (PBS) was given during seven postoperative days. Hereupon, subconvulsive dose of lindane (4 mg/kg, i.p.) was administered and behavioral features of seizures were observed alongside with EEG recordings. *Results*: Our data showed that the incidence and severity of lindane-induced seizures was significantly higher in the CP/CPPS-PBS group than in the Sham-PBS group. CORM-A1 treatment significantly decreased seizure incidence, prolonged seizure latency, and reduced seizure severity in CP/CPPS rats compared to vehicle treatment (CP/CPPS-CORM vs. CP/CPPS-PBS). Also, CORM-A1 treatment significantly reduced the number and duration of ictal periods induced by lindane in CP/CPPS animals compared to vehicle treatment. *Conclusions*: It could be concluded that CORM-A1 treatment reduced both behavioral and EEG signs of increased seizure susceptibility in rats with CP/CPPS, thus it could be a potential therapeutic target.

## 1. Introduction

Chronic prostatitis and chronic pelvic pain syndrome (CP/CPPS) represent significant clinical challenges due to their high prevalence, complex etiology, and notable impact on quality of life. CP/CPPS is characterized by persistent pain or discomfort in the pelvic region lasting at least three of the past six months, typically accompanied by lower urinary tract symptoms such as increased urinary frequency, nocturia, dysuria, urgency, weak stream, a sensation of incomplete bladder emptying, and pelvic discomfort [1,2].

Recent studies have shown that CPPS is not confined to peripheral pathology but also involves central nervous system (CNS) alterations [3,4]. Neuroinflammation plays a pivotal role in the pathogenesis of CP/CPPS [5]. Chronic pelvic inflammation induces biochemical changes in sensory nerve fibers, disrupting the functions of the immune, nervous, and endocrine systems, which contributes to peripheral sensitization [6]. This sensitization leads to further changes in the CNS, including altered synaptic plasticity, ultimately resulting in central sensitization and increased pain perception [7,8]. Additionally, neuroinflammation sustains chronic pelvic pain through activation of glial cells, the release of proinflammatory cytokines, and disturbances in neurotransmitter homeostasis [9].

Beyond lowering pain thresholds and perpetuating chronic pain, neuroinflammation has also been implicated in increasing susceptibility to epileptic seizures by promoting abnormal electrical activity in neurons [10,11]. Brain inflammation is initiated by the activation of microglia and astrocytes, which release proinflammatory cytokines such as interleukin (IL)-1β, tumor necrosis factor (TNF)-α, and IL-6. These cytokines contribute to neuronal hyperexcitability and epileptiform discharges [12,13]. Furthermore, neuroinflammation can compromise the integrity of the blood-brain barrier (BBB), facilitating the entry of inflammatory molecules into the brain and sustaining epileptic activity [14]. In our previous study, we observed that rats with CP/CPPS had reduced seizure threshold and increased susceptibility to lindane-induced seizures by elevating the level of IL-1β and IL-6 in certain brain regions like hippocampus, cortex and thalamus. This suggests that CP/CPPS may elevate seizure risk via neuroinflammatory mechanisms within the CNS [15].

On the other hand, carbon monoxide-releasing molecules (CORMs) are compounds capable of releasing controlled amounts of carbon monoxide (CO) in biological systems and have gained interest due to their anti-inflammatory and neuroprotective properties [16,17]. CORMs have been reported to attenuate oxidative stress and exert anxiolytic effects [18], modulate inflammatory responses, reduce proinflammatory cytokine release [19]. It is also reported that CORM-A1 may act protectively in models of neonatal seizures by attenuating oxidative stress-induced neurovascular damage [20]. Additionally, CORM-A1 effectively prevented seizure-induced impairments in cerebrovascular responses when administered before or during seizures [21] suggesting that CORMs could modulate susceptibility to seizures in inflammatory states, like in CP/CPPS.

Having in mind these considerations, following mechanistic sequence could be hypothesized: CP/CPPS induces persistent peripheral nociceptive input, which promotes neuroinflammation and microglial activation. This inflammatory milieu may disrupt the excitatory—inhibitory balance, thereby reducing seizure threshold and increasing susceptibility to pronconvulsive stimuli. In this context, we hypothesized that CORM-A1 could interrupt this cascade by attenuating neuroinflammation and restoring neuronal homeostasis. However, this has not been investigated yet In this study we tested this hypothesis using both behavioral seizure outcomes and EEG measurements following lindane challenge.

Having this hypothesis in mind, the aim of our current study was to examine the therapeutic potential of CORM-A1 in modulating susceptibility to lindane—induced seizures in rats with CP/CPPS through behavioral and electroencephalographic (EEG) study.

## 2. Materials and Methods

### 2.1. Animals and Ethical Statement

Male Wistar Albino rats weighing between 250 and 300 g (total n = 32) were used in this study. The animals were maintained in a controlled environment with a temperature of 22 ± 2 °C, relative humidity of 50 ± 5%, and a 12 h: 12 h light: dark cycle, with lights on from 8:00 a.m. Throughout the study, the rats had unlimited access to standard food and water. Animals’ welfares were constantly monitored. Exclusion criteria were appearance of any signs of behavioral or physical impairments. There were no dropouts throughout the experiments. Experimental unit was a single animal. All animals were coded, and experimenters were blinded to its group allocation.

All experimental procedures were approved by the local Ethics Committee authority (Approval No. 323-07-01339/2017-05/3) and fully in accordance with national and international standards in laboratory animal welfare.

### 2.2. Experimental Design

Upon laboratory acclimatizing of seven days, EEG electrodes were implanted to rats five days prior to the intraprostatic injection. Depending on the intraprostatic injection, total number of rats was firstly randomly divided into two cohorts using a randomization table. In the first cohort, CP/CPPS has been induced by intraprostatic 3% λ-carrageenan injection (CP/CPPS, n = 16 rats), while another cohort was Sham operated rats (intraprostatic 0.9% NaCl injection at time of surgery, Sham, n = 16 rats). Afterwards, one half of rats from each of these cohorts were administered CORM-A1 (2 mg/kg/day, i.p. for seven consecutive days upon intraprostatic injection). Phosphate-buffered saline (PBS, 0.1 mL/kg/day, i.p,) was given as the solvent to the other half of rats. Thus, the Sham-PBS, Sham-CORM, CP/CPPS-PBS, CP/CPPS-CORM groups were formed (n = 8 per each group). Animals were randomly assigned to treatment groups using a randomization table.

**Figure 1:**
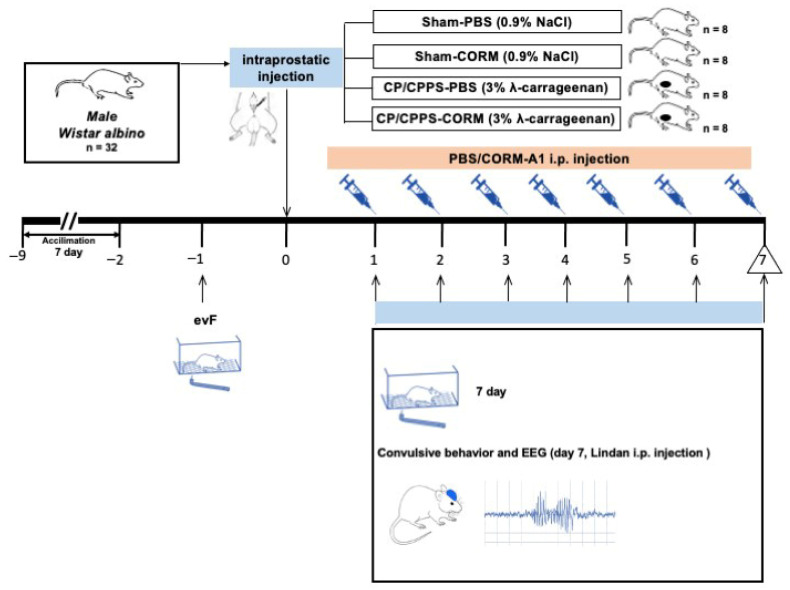
Experimental design. To evaluate CORM treatment and convulsion responses together, the following four experimental groups were formed from each experimental arm: 1: Sham-PBS, 2: Sham-CORM, 3: CP/CPPS-PBS, 4: CP/CPPS-CORM. NaCl was applied intraprostatically to the Sham groups, and 3% λ-carrageenan solution was applied to the CP/CPPS groups. CORM-A1 (2 mg/kg/day) was administered intraperitoneally (i.p.) to the CORM groups every day from the 1st day to the 7th day after surgery. Convulsive tests were conducted to assess convulsive behavior and EEG responses, by administering subconvulsive dose of 4 mg/kg lindane i.p. to all groups on the 7th day. Pain threshold was measured on the—1 and 7th days.

To evaluate convulsive behaviors and EEG responses, convulsion tests were performed by administering a sub-convulsive dose of 4 mg/kg lindane i.p. to all groups on the 7th postoperative day. Mechanical pain threshold was evaluated using the evF device on days −1 and 7 (in reference to intraprostatic injection, surgical operation) (Figure 1). All assessments, including seizure–related behavioral and EEG recordings, were conducted by investigators blinded to the group allocation.

### 2.3. Placement of Electrodes for EEG Recording

Electrodes for bipolar EEG registration were implanted five days prior to the intraprostatic injection to ensure proper recovery period for the animals. Before surgery, the surgical site was disinfected, and anesthesia was induced using sodium thiopental (40 mg/kg, i.p.). Using a stereotaxic apparatus, three gold-plated electrodes were positioned in specific cortical regions based on the Paxinos and Watson (2006) brain atlas: frontal cortex (2 mm anterior to bregma and 2 mm lateral to the midline), parietal cortex (2 mm anterior to lambda and 2 mm lateral), and occipital cortex (2 mm posterior to lambda) [22]. The electrodes were secured with dental acrylic and attached to the recording connector. Animals were further acclimated to the EEG recording setup 24 h prior to each data acquisition.

### 2.4. Experimental CP/CPPS Model Development and Surgical Procedure

In this study, the CP/CPPS model was developed as per previously reported protocols [20,23,24]. For general anesthesia, rats were administered sodium thiopental (40 mg/kg, i.p.). Hereupon, the scrotum and lower abdomen were sterilized, and the rats were placed in a supine position on a heating pad. After local anesthesia, a 1–1.5 cm vertical incision in the midline of the lower abdomen was performed and the ventral prostate lobes were exposed. 25 μL of 3% λ-carrageenan was injected into both prostate lobes of the animals in the CP/CPPS group, while 0.9% NaCl was given to the sham group.

### 2.5. Scrotal Pain Sensitivity Assessments: Mechanical Pain Threshold

To assess development of mechanical allodynia and progression of experimental CP/CPPS, the scrotal pain threshold was measured before (one day, −1) and after the intraprostatic injection (postoperative day 7). An electronic von Frey anesthesiometer (eVF) was used for these evaluations. Prior to testing, animals were acclimated to plexiglas chambers twice daily for 15–20 min and allowed 30 min of habituation during the measurement session. The eVF probe was applied perpendicularly to the scrotal skin, and pressure gradually increased until a withdrawal reflex was observed. The highest pressure recorded at the reflex response was noted as the pain threshold. Each animal’s threshold was calculated as the mean of three consecutive measurements. All evF measurements were performed by the same trained experimenter at a fixed time each day to minimize diurnal variability. Upon testing, the rats were returned to their home cages.

### 2.6. Behavioral and EEG Characteristics of Convulsive Behavior

To evaluate seizure susceptibility of rats subconvulsive dose of lindane (4 mg/kg, intraperitoneally) dissolved in dimethyl sulfoxide (DMSO) was administered to the animals on the seventh day after surgery. Lindane dosage has been chosen based on prior studies on dose-response findings demonstrated that lindane dose of 4 mg/kg produces low seizure incidence in control animals [25]. Pilot experiments in our current settings confirmed that this dose produces near—threshold seizure susceptibility under current conditions Convulsive behavior and EEG were recorded to evaluate brain hyperexcitability in these rats depending on the previous treatment.

Behavioral signs of seizures (i.e., convulsive behavior) were monitored for 30 min following Lindane administration. During this period, seizure incidence, latency to seizure onset, and seizure severity were assessed. Seizure incidence was calculated as the percentage of animals exhibiting seizures relative to the total number in each group. The latency period was defined as the time elapsed from lindane injection to the appearance of the first seizure sign; animals that did not develop seizures were assigned a latency of 30 min. Seizure severity was rated using a 0-to-4 scale [26]: 0—no seizures; 1—head shaking and/or jaw contractions; 2—myoclonic jerks or “kangaroo posture” (standing on hind limbs with forelimb clonus); 3—generalized clonic seizures involving tonic contractions of forelimbs, hind limbs, and tail; 4—severe clonic-tonic seizures lasting more than 20 s or recurrent seizures lasting over 5 min (status epilepticus).

Bipolar EEG recordings from freely moving rats were obtained using an 8-channel system (RIZ, Zagreb, Croatia). Signal acquisition was carried out via a 16-bit SCB-68 interface (National Instruments, Austin, TX, USA). Electrode impendence was checked prior to recording and impendence <10 kΩ were acceptable. Each animal was placed in a transparent plexiglas chamber (Elunit, Belgrade, Serbia) and connected to the EEG apparatus through a flexible tether, allowing for simultaneous behavioral monitoring during the recording period. Following Lindane administration, EEG activity was recorded for 30 min, after which the animals were returned to their cages. Data was sampled at 512 Hz. To minimize electrical interference, a 50 Hz notch filter, along with a 0.3 Hz high-pass and 100 Hz low-pass filter, was applied. Continuous EEG recordings were captured using NeuroSciLaBG software (version 1.2.1), a LabVIEW-based program developed in-house.

EEG data were initially examined visually to identify seizure-related activity. Screening for ictal periods and their identification were done by an evaluator blinded to group allocation. Subsequent quantitative analysis was conducted using NeuroSciLaBG software (Belgrade, Serbia). Ictal period was defined based on specific criteria indicative of lindane-induced epileptiform discharges [27], including spontaneous, widespread rhythmic spike-wave patterns; durations of at least one second; and wave amplitudes at least double the baseline EEG. For each subject, the total number and duration of ictal periods during the 30-min recording period were documented. EEG analysis in this study was focused on number and duration of ictal periods because these masseurs most directly reflect seizure susceptibility under acute lindane challenge. Quantitative spectral analysis was not included due to specific design of post-lindane recording window optimized for seizure detection rather than spectra characterization.

### 2.7. Substances

All chemicals of analytical grade used in this study, including PBS, DMSO, λ-carrageenan, CORM-A1, and Lindane, were obtained from Sigma Aldrich (St. Louis, MO, USA). Fresh solutions were prepared immediately prior to each administration. This is of particular importance for CORM-A1 solution.

### 2.8. Statistics

No missing data were spotted in this study as all rats completed the intended experimental protocol and their data were included in the final analysis. To test the normality of the data we used Kolmogorov-Smirnov test. Statistical significance of differences in seizure incidence was assessed by Fisher’s test. The statistical significance of differences in latency period, seizure intensity, ictal periods number and duration between groups were evaluated with the Kruskal-Wallis test and the Mann-Whitney post hoc test having in mind non-parametric distribution. Data were presented as medians with interquartile ranges. Significance of between groups differences in pain threshold were assessed using ANOVA with Tukey’s post hoc test for multiple comparisons. Significance of intra-group comparisons were assessed using the t test for repeated measurements. Data were expressed as mean ± SD. Statistical approach has been in line with Statistical strategies in other reports [28]. Statistical significance: *p* < 0.05 and *p* < 0.01.

## 3. Results

### 3.1. Effect of CORM-A 1 on Scrotal Pain Threshold

Scrotal pain threshold was measured before (day −1) and after surgery (day 7) in all groups. Baseline thresholds showed no significant differences among rats (*p* > 0.05). Pain thresholds remained stable in Sham groups throughout (*p* > 0.05). However, in CP/CPPS groups, the threshold dropped significantly on postoperative day 7 (*p* < 0.001), compared both to Sham groups and pre-surgery levels. Notably, CORM-A1 treatment in the CP/CPPS-CORM group significantly raised pain thresholds compared to CP/CPPS-PBS (*p* < 0.001). CORM-A1 had no significant effect in Sham animals (*p* > 0.05) (Table 1).

### 3.2. Effect of CO Modulation on Convulsive Behavior

**Figure 2:**
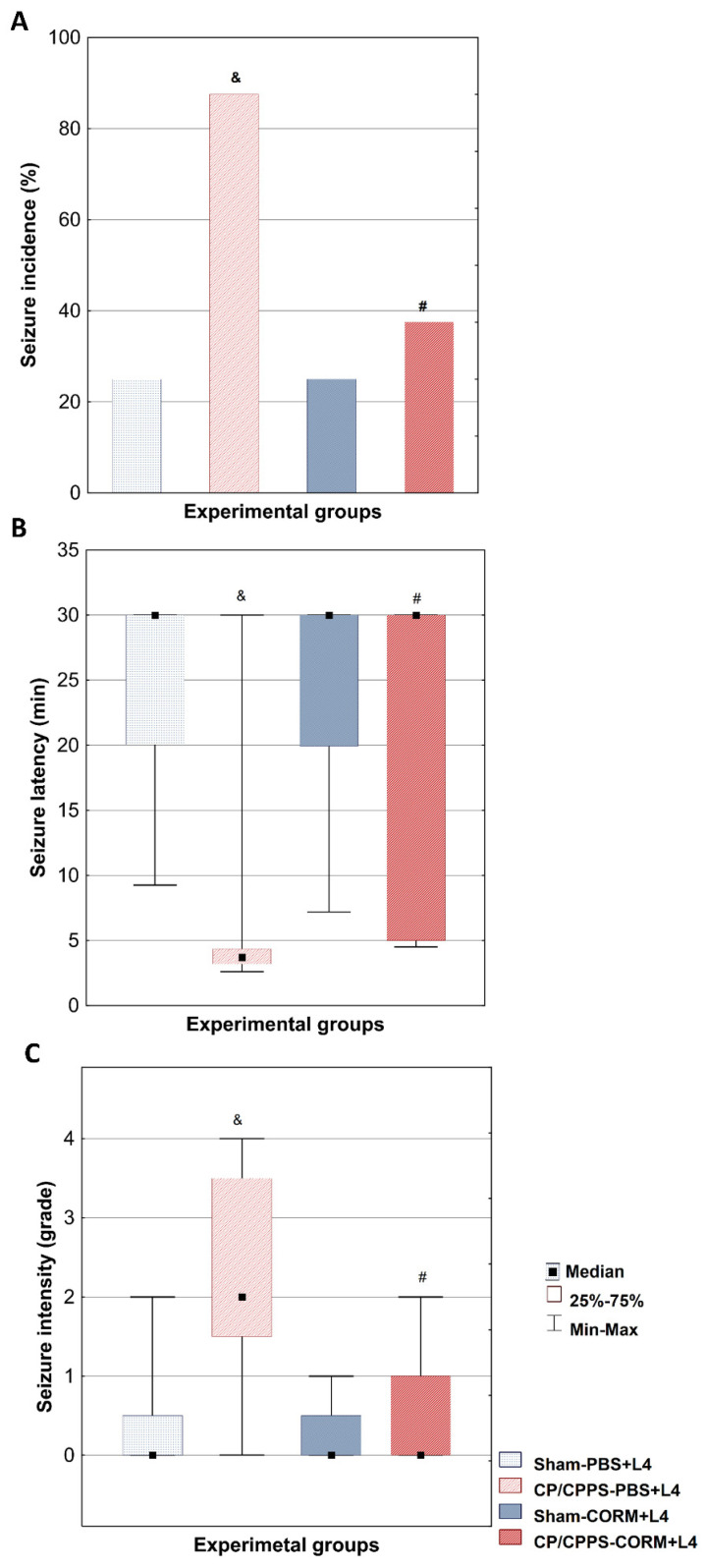
Effect of CORM-A1 on convulsive behavior evoked by subconvulsive dose of lindane in rats with CP/CPPS. On the seventh day after surgery, subconvulsive dose of lindane (4 mg/kg) was administered intraperitoneally to rats to evaluate their susceptibility to seizures. Seizure incidence (**A**) was expressed as as a percentage (%) of the animals with convulsions. The latent period (**B**) was defined as the time from the injection of lindane to the appearance of the first observable convulsion sign. The intensity of seizures (**C**) was assessed on a descriptive scale with grades from 0 to 4. Statistical significance of differences in seizure incidence was assessed by Fisher’s test. The statistical significance of differences in latent period and seizure intensity between groups were evaluated with the Kruskal-Wallis test and the Mann-Whitney post hoc test (^&^ *p* < 0.05 vs. Sham-PBS group; ^#^ *p* < 0.05 vs. CP/CPPS-PBS group). For details and abbreviations see captions in Figure 1.

The seizure incidence after a subconvulsive lindane dose was significantly higher in the CP/CPPS-PBS group (87.5%) compared to the Sham-PBS group (25%) (*p* < 0.05). In animals treated with CORM-A1, seizure incidence was 25% in the Sham-CORM group and 37.5% in the CP/CPPS-CORM group, with no significant difference between them (*p* > 0.05). Actually, CORM-A1 treatment significantly reduced seizure incidence in CP/CPPS animals compared to PBS-treated CP/CPPS controls (CP/CPPS-CORM vs CP/CPPS–PBS, 37.5% vs. 87.5%, *p* = 0.0455, Figure 2A).

Analysis of the latency period until the first convulsive seizure was significantly shorter in the CP/CPPS-PBS group compared to the Sham-PBS group (*p* < 0.05, Figure 2B). In addition, postoperative treatment with CO donor in prostatitis animals was shown to significantly prolong the median time to the first seizure compared to solvent-treated prostatitis animals (CP/CPPS-CORM vs. CP/CPPS-PBS, *p* = 0.0462, Figure 2B).

The seizure severity induced by lindane was significantly higher in the animals in the CP/CPPS-PBS group compared to the animals in the Sham-PBS group (*p* < 0.05, Figure 2C). In addition, the maximum seizure score observed in the CP/CPPS-PBS group was 4, while this value was 2 in the Sham-PBS group. Postoperative CORM-A1 treatment applied to the animals in the CP/CPPS-CORM group provided a statistically significant decrease in seizure severity compared to the animals in the CP/CPPS-PBS group (*p* = 0.0408, Figure 2C).

In the Sham-PBS group, 75% of animals showed no seizures (grade 0), while grades 1 and 2 occurred equally at 12.5%, and no grade 3 or 4 seizures were observed. In the CP/CPPS-PBS group, seizures were more severe: 25% had grade 4, 37.5% had grade 2, and grades 0, 1, and 3 each at 12.5%. Sham-CORM animals mainly had grades 0 (75%) and grade 1 (25%) seizures, with no higher grades. CP/CPPS-CORM animals mostly had grade 0 (62.5%), with grade 1 (25%) and 2 (12.5%) seizures; no grade 3 or 4 seizures occurred (Table 2). Postoperative CO donor treatment (CORM) in CP/CPPS animals significantly increased the frequency of grade 0 seizures compared to CP/CPPS-PBS (*p* < 0.05).

### 3.3. Effect of CORM-A1 on EEG Ictal Activity Induced by Lindane in Animals with CP/CPPS

**Figure 3:**
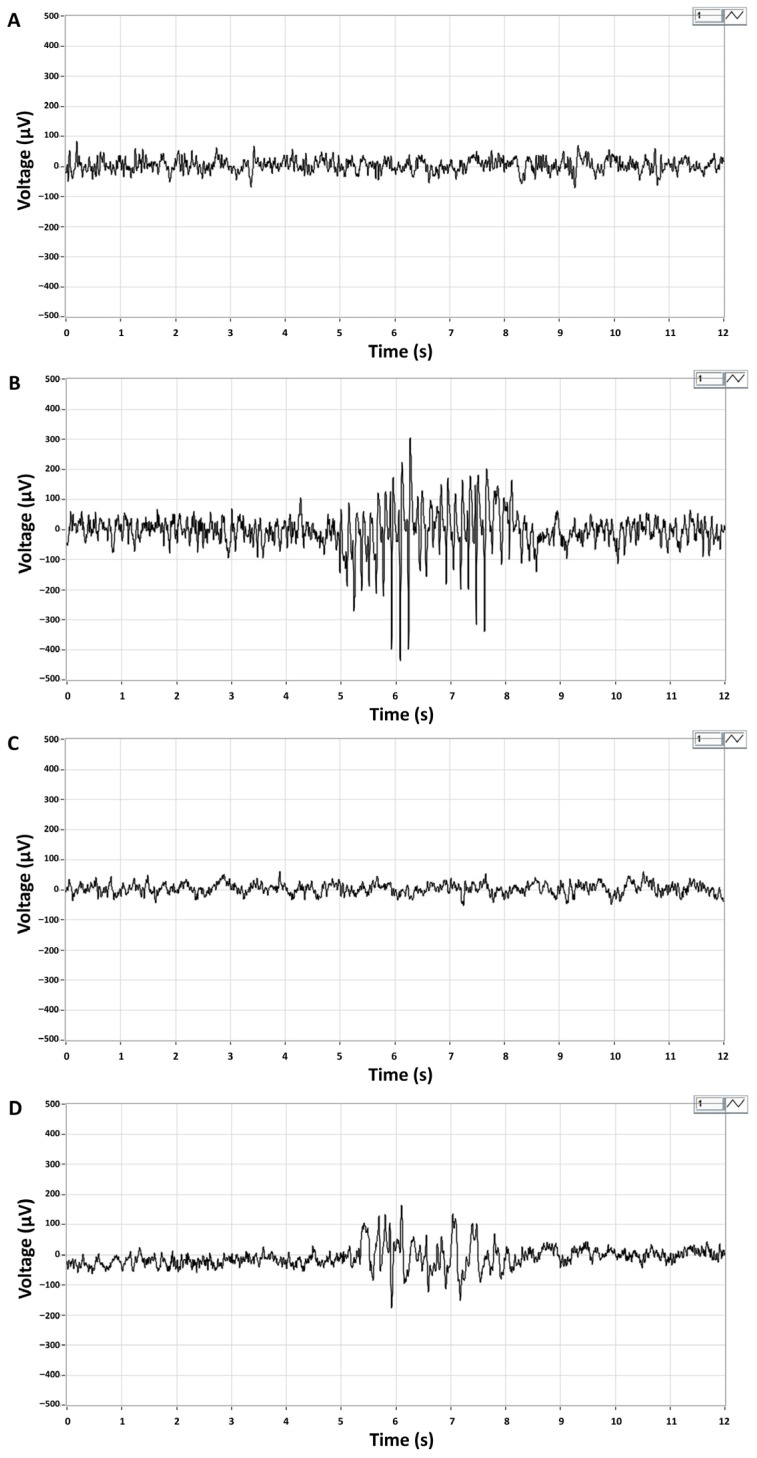
Representative EEG recordings of brain activity in Sham-PBS (**A**) and Sham-CORM (**C**) animals, and ictal EEG periods in CP/CPPS-PBS (**B**) and CP/CPPS-CORM (**D**) animals. On the 7th postoperative day, animals received an intraperitoneal injection of a subconvulsive dose of lindane (4 mg/kg, i.p.), and EEG activity was recorded for 30 min. Ictal periods are characterized by a series of high-amplitude spikes (200–300 μV) with a dominant frequency of 7–8 Hz in the alpha band. Recording leads: right frontal—left parietal. For details and abbreviations see caption in Figure 1.

Representative ictal periods showed high-amplitude spike series in CP/CPPS-PBS animals and lower amplitude and frequency spikes in CP/CPPS-CORM animals during the 30-min EEG recording upon lindane administration in subconvousive dose (4 mg/kg, i.p.) (Figure 3).

**Figure 4:**
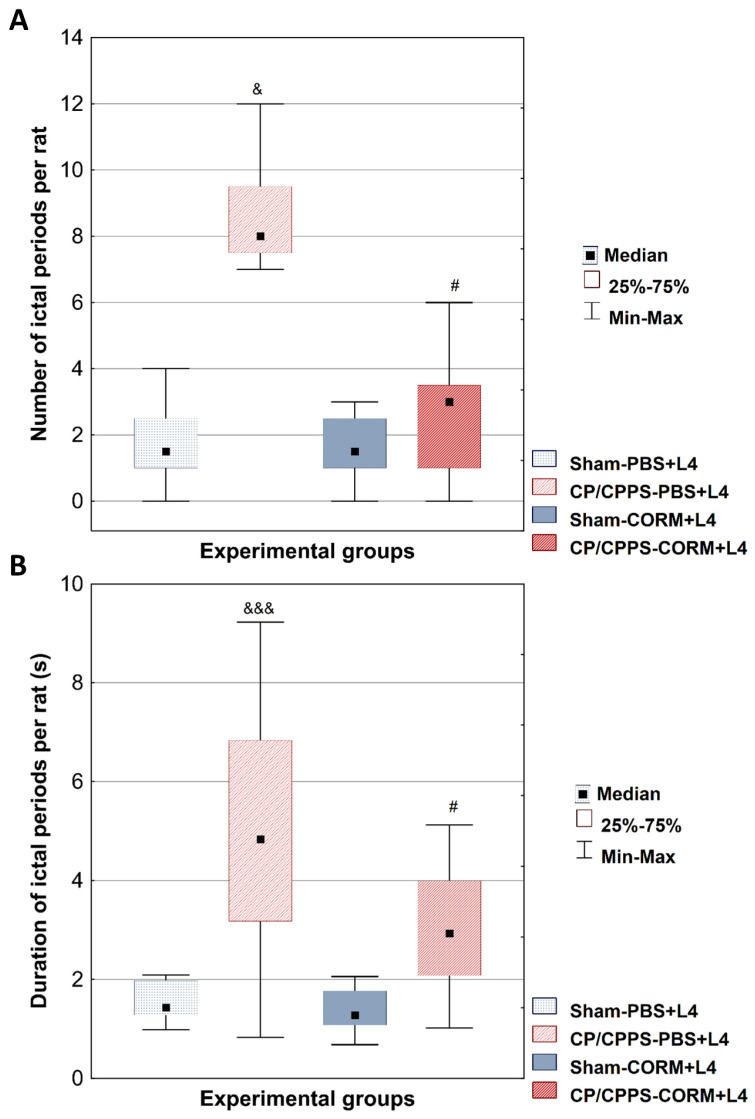
Effect of CORM-A1 on the number (**A**) and duration (**B**) of EEG ictal periods per animal induced by a subconvulsive dose of lindane in rats with CP/CPPS. The number (**A**) and duration (**B**) of EEG ictal periods per rat was determined for all four groups. Statistical significance of differences between groups was assessed using the Kruskal-Wallis test with Mann-Whitney post hoc test (^&^ *p* < 0.05; ^&&&^ *p* < 0.05 vs. Sham-PBS; ^#^ *p* < 0.05 vs. CP/CPPS-PBS). For details and abbreviations see caption in Figure 1.

The number of EEG ictal periods was significantly higher in the CP/CPPS-PBS group than in the Sham-PBS group (*p* < 0.05, Figure 4A). Postoperative CORM-A1 treatment significantly reduced the number of EEG ictal periods compared to PBS treatment in animals with prostatitis (*p* = 0.0239, Figure 4A).

Similar results were found in the analysis of EEG ictal period duration. The duration was significantly longer in the CP/CPPS-PBS group compared to Sham-PBS (*p* < 0.001, Figure 4B). Postoperative treatment with the CORM-A1 significantly reduced ictal period duration in CP/CPPS animals (CP/CPPS-CORM vs. CP/CPPS-PBS, *p* = 0.0203, Figure 4B).

## 4. Discussion

In our study, we aimed to evaluate therapeutic potential of CORM-A1, chemical compound that slowly releases carbon monoxide gas, in amelioration of seizure susceptibility in CP/CPPS rats. Our data showed that the incidence of lindane-induced seizures was significantly higher in the CP/CPPS-PBS group (87.5%) than in the Sham-PBS group (25%). This is in agreement with our previous study showing increased seizure susceptibility in CP/CPPS rats compared to corresponding Sham controls [15]. CORM-A1 treatment significantly decreased seizure incidence, prolonged seizure latency and reduced seizure severity in CP/CPPS rats comparing to vehicle treatment (CP/CPPS- CORM vs. CP/CPPS-PBS, Figure 2). Actually, seizures were generally mild (grade 0–1) in the Sham-PBS and Sham-CORM groups and no high-severity seizures were observed (Table 2), while seizure severity increased significantly in the CP/CPPS-PBS group and the maximum score reached 4. CORM-A1 treatment reduced seizure severity in the CP/CPPS group to levels close to the sham groups. Thus, CORM-A1 ameliorated seizure susceptibility induced by CP/CPPS in rats.

EEG analyses in our study also supported findings from convulsive behavior monitoring. Ictal activity was low in terms of number and duration of ictal periods in the sham groups upon lindane chalange. On the contrary, the number and duration of ictal periods induced by lindane increased significantly in the CP/CPPP rats on vehicle treatment (CP/CPPS + PBS). However, CORM-A1 treatment significantly reduced these parameters in CP/CPPS animals compared to vehicle treatment (Figure 4). Thus, these results speak in favor of CORM-A1 beneficial effects on seizure susceptibility induced by CP/CPPS in rats.

### 4.1. Possible Mechanisms

In our previous study [15], we have demonstrated that increased susceptibility to seizures in rats with CP/CPPS is associated with increased level of proinflammatory cytokines IL-1β and IL-6 in brain regions like the cerebral cortex and thalamus, thus indicating that CPPS-induced neuroinflammation is a possible mediator of increased seizure susceptibility in these rats.

Moreover, further studies investigating the relationship between CP/CPPS and neuroinflammation have shown that CP/CPPS creates a neuroinflammatory environment in the CNS [27,29] Significant increase in the expression of inflammatory genes and cytokines have been demonstrated in CP/CPPS patients [30]. Also, microglial activation at the spinal cord level has been reported to increase the expression of proinflammatory cytokines such as IL-1β and CCL3, as well as neuromodulators including the P2X4 receptor and BDNF in experimental autoimmune prostatitis models [31].

It is now well-known that neuroinflammation disrupts the balance between inhibition and excitation within neural circuits, facilitates seizure formation, and promotes the consolidation of epileptic networks [32]. Microglial activation is proposed to play a key role in the onset of seizures by promoting the release of proinflammatory cytokines such as IL-1β and TNF-α [33]. Namely, the activation of P2X4 receptors on microglia leads to the release of BDNF, which binds to TrkB receptors on neurons, downregulating KCC2 expression. This reduction impairs GABAergic and glycinergic inhibition, resulting in increased neural excitability [34]. Thus, microglial and astrocyte activation observed in CP/CPPS may similarly increase neural excitability via inflammatory cytokines and neuromodulators [35] what is congruent with our current and previous findings [14]. On the other hand, CO-releasing molecules has been demonstrated to reduce neural excitability by blocking P2X4 receptors [36]. Considering these data, the effect of CORM-A1 in reducing seizure severity of CP/CPPS rats might be due to suppression of microglial activation and related neuroinflammation by inhibiting the activation of P2X4 receptors.

Zheng et al. (2019) showed that CORM-3 administration after spinal cord injury inhibited inflammasome activation and reduced pyroptosis, which is inflammation-associated cell death [37]. Lin et al. (2017) reported that CORM-3 suppressed IL-1β-induced inflammatory responses by increasing HO-1 expression in rat brain astrocytes [38]. Bani-Hani et al. (2006) reported that CORM-3 significantly reduced inflammatory responses in microglia cells [39]. Wang et al. (2018) showed that CORM-3 reduced neuronal damage and microglia activation and showed neuroprotective effect by protecting the blood-brain barrier in a transient middle cerebral artery occlusion model [11]. These studies, consistent with our findings, support that CORMs provide neuroprotective effects.

These CORMs effects could be the consequence of their role in redox homeostasis. It is known that oxidative stress plays an important role in the pathophysiology of epilepsy, as well as that ROS and inflammation are mutually reinforcing processes in epileptogenesis [40]. On the other hand, it was shown that CORM-A1 molecules reduce oxidative stress and improve mitochondrial functions through Nrf2 activation [41] since Nrf2 transcription factor protects cells against oxidative stress by activating antioxidant genes and has a neuroprotective effect in both human and animal studies [42]. It was reported that CORM-2 increased the expression of heme oxygenase-1 (HO-1) by activating the Nrf2/ARE pathway and thus reduced TNF-α-induced inflammation. As a result, CORM-2 protected cells against oxidative stress by suppressing inflammation [43]. Also, CORM-3 was shown to reduce oxidative stress-induced cell damage and protect mitochondrial functions via the activation of the Nrf2/HO-1 pathway [44]. Actually, HO-1/CO system acts as an active intracellular defense mechanism in epileptic cells and provides a compensatory response to oxidative damage during epilepsy [10]. These studies suggest that activation of the Nrf2 pathway can provide both neuroprotective effects and slow down seizure progression by suppressing processes such as glial activation, inflammation, and mitochondrial dysfunction; in this context, the antioxidant and anti-inflammatory effects of CORMs via the Nrf2/HO-1 pathway support their potential as treatment candidates in epilepsy.

### 4.2. Limitations

Our study has several limitations. The use of a single, near-threshold dose of lindane (4 mg/kg) was based on prior dose-response studies demonstrating low seizure incidence in control animals. Pilot experiments in our laboratory confirmed that this dose produced minimal seizures in sham rats under our specific experimental conditions. Nonetheless, reliance on a single dose of lindane and time point limits our ability to generalize the findings to other seizure models, which should be explored in future work. In order to elucidate the exact mechanisms of herein observed outcomes in reducing seizure susceptibility in a model of CP/CPPS, further studies are needed with special emphasis on direct effects in the CNS, having in mind recent findings on direct effects on ion channels and CORMs properties (PMID 29500353). Further studies should also include spectral characterization of recorded EEG activity.

## 5. Conclusions

In our study, CORM-A1 significantly reduced severity of lindane-induced seizures in CP/CPPS rats, as well as number and duration of ictal periods in EEG, thus it reduces susceptibility to seizures in rats with CP/CPPS. CORM-A1 may be a potential therapeutic agent in reducing the brain hyberexcitability in model of CP/CPPS.

## Figures and Tables

**Table 1:** Scrotal pain threshold in experimental groups.

Groups	Scrotal Pain Threshold (mN)	
	Day −1	Day 7
Sham-PBS	698.61 ± 49.58	672.72 ± 53.08
Sham-CORM	680.81 ± 51.44	692.12 ± 41.98
CP/CPPS-PBS	732.15 ± 77.76	250.04 ± 34.36 ***^, ###, +++^
CP/CPPS-CORM	742.77 ± 83.55	386.84 ± 68.07 ***^, &&&^

Scrotal pain threshold was tested using eVF aesthesiometer once before (day −1) and one after the surgery (day 7). Data are expressed as mean ± SD. Significance of between groups differences (^&&&^ *p* < 0.001 vs. Sham-PBS; ^+++^ *p* < 0.001 vs. Sham-CORM; ^###^ *p* < 0.001 vs. CP/CPPS-PBS) were assessed using ANOVA with Tukey’s post hoc test for multiple comparisons. Significance of intra-group comparisons (*** *p* < 0.001, vs. −1) were assessed using the *t* test for repeated measurements. For groups see caption to Figure 1.

**Table 2:** Effect of CORM-A1 on the distribution of seizure grades induced by a subconvulsive dose of lindane in rats with CP/CPPS.

Groups	Grade Distribution (%)				
	G0	G1	G2	G3	G4
Sham-PBS	75.0 ^#^	12.5	12.5	0	0
Sham-CORM	12.5 ^&^	12.5	37.5	12.5	25.0
CP/CPPS-PBS	75.0	25.0	0	0	0
CP/CPPS-CORM	62.5	25.0	12.5	0	0

The percentage representation of each seizure grade within the groups is shown. Statistical significance of differences between groups in the distribution of seizure grades was assessed using Fisher’s exact test (^&^ *p* < 0.05 vs. Sham-PBS; ^#^ *p* < 0.05 vs. CP/CPPS-PBS). For details and abbreviations see caption in Figure 1.

## Data Availability

The raw data supporting the conclusions of this article will be made available by the authors on request.

## Abbreviations

CORMs	CO-releasing molecules
CO	Carbon monoxide
EEG	Electroencephalography
CNS	Central nervous system
